# PROGRAM ADAPTATIONS FOR DIVERSE COMMUNITIES IN BEST PROGRAMS FOR CAREGIVING

**DOI:** 10.1093/geroni/igae098.0034

**Published:** 2024-12-31

**Authors:** Megan Huth, Sara Powers, Zoe Fete, Rachel Cannon, Morgan Minyo, David Bass

**Affiliations:** Benjamin Rose, Cleveland, Ohio, United States; Benjamin Rose, Cleveland, Ohio, United States; Benjamin Rose, Cleveland, Ohio, United States; Benjamin Rose, Cleveland, Ohio, United States; Benjamin Rose Institute on Aging, Cleveland, Ohio, United States; Benjamin Rose, Cleveland, Ohio, United States

## Abstract

To better understand the role of cultural adaptations among evidence-based dementia caregiving programs included on Best Programs for Caregiving (BPC), online surveys were distributed and collected from organizations delivering BPC programs in the community in 2023. Surveys collected data on: 1) Implementation of a culturally adapted program, 2) Diverse groups the program was adapted for, (e.g., racial and ethnic groups, LGBTQ+ groups), and 3) Types of cultural adaptations made (e.g., marketing materials revised, program delivered in language, consultation with diverse group members). Results indicated that 23.2% of organizations delivering BPC programs made adaptations for diverse groups, most commonly: 1) Hispanic/Latino (61.5%), 2) LGBTQ+ (53.8%), 3) Black/African American (46.2%). Among the programs that made adaptations, 100% consulted with group members or experts on the diverse group, 92.8% adapted content for members of the group, 53.8% provided staff training and began delivering the program in the group’s language, and 46.2% created translations of the materials and adapted marketing and outreach tools for the diverse group. The least common adaptation was conducting research to show that the program benefited the diverse groups (25%). These findings show that while a larger than expected number of BPC have made modifications for diverse groups, the majority of programs (76.8%) were not adapted for any diverse community. The discussion will center on the importance of developing culturally adapted programs that better cater to the needs of diverse family and friend caregivers.

